# Evaluating the satisfaction and utility of social networks in medical practice and continuing medical education

**DOI:** 10.1186/s12909-024-05149-z

**Published:** 2024-02-23

**Authors:** Marion Bendayan, Claire Bonneau, Mai Thi Delespierre, Emine Sais, Fanie Picard, Laura Alter, Florence Boitrelle, Laure Cazabat

**Affiliations:** 1 Service de Biologie de la Reproduction– Andrologie– CECOS, Centre Hospitalier Intercommunal de Poissy Saint Germain en Laye, 10 rue du Champ Gaillard, 78300 Poissy, France; 2 INRAE, ENVA, BREED, UVSQ, Université Paris Saclay Jouy-en-Josas, Gif-sur-Yvette, France; 3 Département de chirurgie, Institut Curie– Saint-Cloud, 35 rue Dailly, 92210 Saint Cloud, France; 4 Inserm U900, Institut Curie, Université Versailles Saint Quentin en Yvelines, Université Paris Saclay, Saint-Cloud, France; 5 Département de Médecine Générale, Faculté de médecine de Montpellier-Nîmes, Nîmes, France; 6 UMR 1198 BREED, équipe RHuMA, UFR Simone Veil Santé, Université Versailles Saint Quentin en Yvelines, Université Paris Saclay, Paris, France; 7 Service de Neurochirurgie, Hôpital Foch, Suresnes, France

**Keywords:** Social network, Continuing medical education, General practitioners

## Abstract

**Background:**

Digital health has surged during the Covid health crisis, and the use of social media, already prevalent in medicine, has significantly increased. There are Social Networks groups dedicated to physicians with an educational purpose. These groups also facilitate peer discussions on medical questions and the sharing of training materials.

**Objectives:**

The aim of our study was to assess the value of these new tools and their contribution to medical education.

**Methods:**

An anonymous questionnaire was conducted among members of a Social Networks community group for physicians. The survey received responses from 1451 participants.

**Results:**

The majority of participants believed they had enriched their medical knowledge and accessed documents they would not have accessed without the group. Subgroup analysis showed that the contribution of this tool is more pronounced for general practitioners and doctors practicing in limited healthcare access.

**Conclusion:**

It is essential to develop digital tools that enhance physician training, and social networks represent a valuable educational tool.

**Supplementary Information:**

The online version contains supplementary material available at 10.1186/s12909-024-05149-z.

## Background

Social Networks are used by over 4 billion people worldwide [1, 2]. Numerous online platforms exist which enable users to share a variety of content including images, documents, and files, as well as to exchange messages. These platforms can be utilized for both personal and professional interactions. The Covid-19 pandemic has significantly accelerated the adoption and integration of digital health technologies. This surge has been driven by social distancing norms and nationwide lockdowns, necessitating people and organizations globally to adapt to new ways of working and living [3]​. The growth in the use of social media in medicine, already prevalent prior to the pandemic, has also significantly increased, becoming a more integral part of medical communication and practice [4]. In medicine, the use of social network for educational purposes is increasingly expanding [5]. Many renowned scientific journals, such as The New England Journal of Medicine and The Lancet, have several Social Networks pages. They frequently share articles or clinical cases with their subscribers.

Governmental health institutions also utilize Social Networks to disseminate information or recommendations as widely as possible [6, 7]. However, these pages are not exclusively for healthcare professionals but aim to reach the broader population with the intent of raising awareness and promoting prevention. On some Social Networks, there are also private community groups, which require membership and access approval by the group’s creators (referred to as administrators). These groups can serve as a tool for medical education.

The most common are groups for medical students who create Social Networks groups with their cohort members and exchange practical information about courses (schedules, locations, etc.), as well as documents, study guides, lessons presentations, and more. The messaging system allows them to comment on shared documents, ask questions, and engage in discussions. However, the majority of these groups consist solely of students, with no interaction with the teaching faculty [5].

A 2019 publication highlighted the benefits of a Social Networks group comprising members of an ophthalmology department, facilitating discussions on service management (on-call shifts, standby duties) as well as sharing lectures and clinical cases [8]. However, this group is restricted to the physicians of the department, and the authors report that its primary use pertains to service management (schedule changes, etc.).

In 2017, a french group was established by two physicians in the Social Network Facebook® (https://fr-fr.facebook.com/groups/240193799818282). This private group is intended for Francophone doctors and medical students. As of September 1, 2022, it has just over 22,000 members. To join, membership requests must be accompanied by the individual’s identity and their place of practice or study.

This Facebook® group facilitates the sharing of experiences among doctors, seeking opinions, disseminating recommendations, and engaging in various discussions on medical or societal topics. A genuine virtual community has formed, with colleagues sharing their daily experiences, anecdotes, and even more personal subjects. Medical opinion inquiries make up the majority of the posts.

The group fosters a participatory medicine approach based on the exchange of information and experiences among professionals and has garnered immense enthusiasm. Numerous doctors have posted messages explaining how this group has transformed their medical practice. Notably, group members frequently highlight the group’s contribution to Continuing Medical Education (CME). CME is defined as a range of educational activities designed to maintain, develop, or increase the knowledge, skills, and professional performance of physicians [9]. According to the International Association for Medical Education, CME includes any activity intended to maintain, develop, or increase the knowledge, skills, and professional capabilities of medical practitioners​ [10]. Indeed, many members regularly provide updates on diseases/treatments/symptoms. They also share a plethora of documents, such as the latest recommendations, scientific articles, etc., to inform and educate the group’s medical community.

In this study, we aimed to assess the use of this Social Networks group and its impact on medical education. To achieve this, we conducted a survey targeting the group’s members, with the objective of understanding the advantages and disadvantages of this group.

## Methods

### Study desing

We conducted a prospective study. An online questionnaire was created using the GoogleForm® online form tool and was shared within the group between June 1, 2022, and July 15, 2022 (Additional Table 1).

The questionnaire was anonymous and was accompanied by a brief explanatory message about the purpose of the study. Responses were voluntary, anonymous, self-reported, and based on willingness to participate. Participants in this survey were clearly informed about the purpose of the study and voluntarily consented to take part.

The study was approved by the Institutional Review Board of Société d’Andrologie de Langue Française (IORG0010678) under reference number 202,207.

The questionnaire underwent a pre-test by 4 physicians from different specialties to assess its format, readability, and comprehension.

The questions were either single-choice or multiple-choice, and some were accompanied by open-ended comments.

It consisted of 27 questions focusing on the group’s contribution in terms of medical pedagogy and Continuing Medical Education (CME).

The first 8 questions concern the demographics of the participants and their use of the group. Questions 9 to 22 concern the medical opinions sought on the group. The majority of these questions involve giving a score between 0 and 10 to various items concerning medical advice taken on the group, considering 0 as “strongly disagree” and 10 as “strongly agree”.

Questions 9, 10, 11, 21, and 22 concern the use of the group and the overall feelings of the participants. Questions 12, 14, 15, and 16 focus on the interest of the platform in medical decision-making and patient care. Questions 13, 17, and 18 assess the impact of the group on the physician’s confidence in their medical decision. Questions 19 and 20 relate to the use of other medical opinion platforms by the participants.

Questions 23 to 26 concern the group’s contribution to CME and these questions involve giving a score between 0 and 10 to various items, considering 0 as “strongly disagree” and 10 as “strongly agree”. Question 23 focuses on the role of the group in maintaining and developing the medical knowledge of its members. Questions 24 and 25 are concerned with the contribution of the platform in terms of bibliographic monitoring and updates on current guidelines. Question 26 pertains to the methods of using the platform.

Question 27 is an open-ended question to allow participants to make any comments they may have.

It should be noted that the term “CME points” was mentioned several times in the questionnaire, which is a term used within the group to describe shared documents such as recommendations from learned societies, scientific articles, etc.

### Statistical analysis

Initially, a descriptive analysis of all the responses to the questions was conducted. Categorical data were presented as the number of participants and percentages.

Subsequently, the association between the profiles of the participants (age, specialty, etc.) and their responses was assessed using the Chi-squared test for categorical variables. Analyses were performed using the R software version 4.1.2. A p-value of < 0.05 was considered statistically significant.

## Results

During the period from June 1, 2022, to July 15, 2022, 1,451 participants responded to at least one question in the questionnaire. The questionnaire was completed in full by 989 participants, i.e. 68,2% of all participants.

### Population characteristics

Among the participants, 83% were female, 63.8% were aged between 30 and 40 years, and 65.1% were general practitioners. Almost all medical specialties were represented, and 59.6% of the participants practiced as independent professionals, while 16.9% were hospital-based, 5.3% were academic hospital-based, and 5.1% were medical residents.

58.5% worked in urban areas, 30.3% in semi-rural areas, and 11.2% in rural areas. Only 28.9% of the participants believed they practiced in a medical underserved area.

A vast majority of the participants (90.7%) logged onto the Social Networks group at a frequency of once a day or more.

**Fig. 1:**
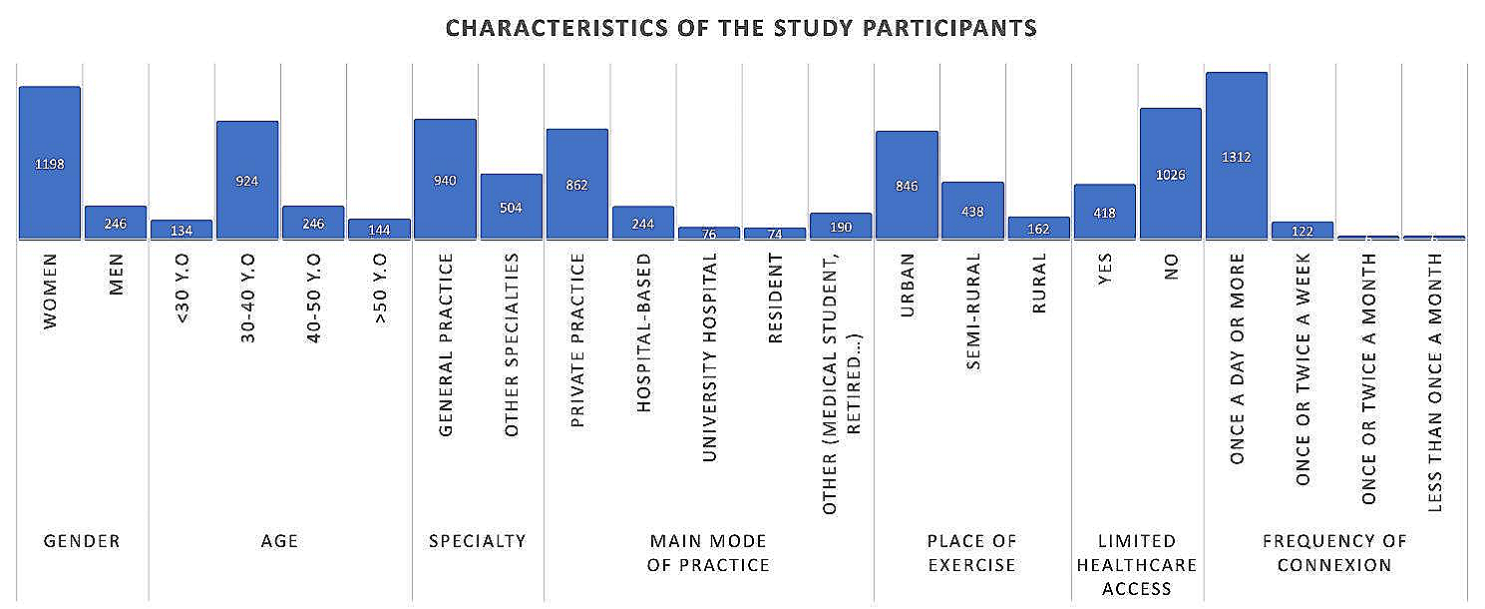
Characteristics of the study participants. The results are presented in the number of participants

Data regarding the population characteristics are illustrated in Fig. 1 and Additional Table 2.

### Descriptive analysis: questions regarding medical opinions

Among the participants, 3.3% reported frequently seeking medical opinions on the group, 25.7% occasionally, 36.3% rarely, and 34.7% never. In total, 65.3% of the participants have sought a medical opinion on the group at least once.

It should also be noted that 594 participants, or 41% of the participants, reported using another platform for medical opinions (either free or paid).

Regarding responding to requests, 8.6% of participants reported frequently responding to medical opinion requests on the group, 36.8% occasionally, 34.3% rarely, and 20.3% never. When asked, “Were the responses provided by your colleagues useful?“, 74.7% of participants gave a score of ≥ 8 on a scale of 0 to 10, considering 0 as “not at all useful” and 10 as “very useful”.

**Fig. 2:**
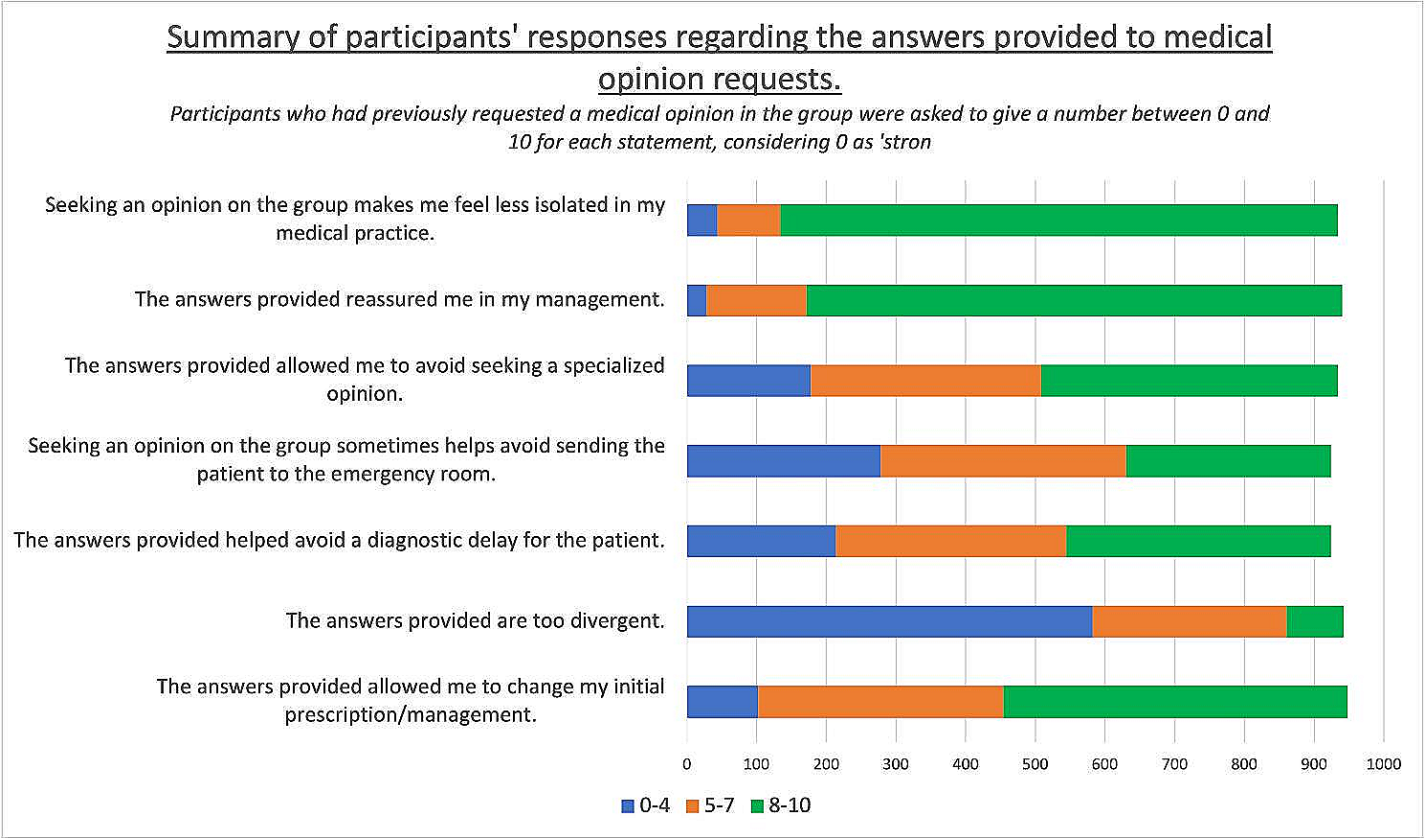
Summary of participants’ responses regarding the answers provided to requests for medical opinions. Participants who had previously sought medical advice in the group were required to give a number between 0 and 10 for each statement, considering 0 as “strongly disagree” and 10 as “strongly agree”. The results are expressed in the number of participants who rated between 0 and 4, between 5 and 7, or 8 and above

Regarding the usefulness of the responses, 81.7% of participants gave a score of ≥ 8 (on a scale of 0 to 10, with 0 being “not at all useful” and 10 being “very useful”) for the statement “The responses provided reassured me in my patient care approach.” and 85.7% for the statement “Seeking opinions on the group makes me feel less isolated in my medical practice.” The results of this question are detailed in Fig. 2 and Additional Table 3.

Regarding the multiple-choice question on the group’s strengths, the “speed of responses” option was chosen by 87.5% of participants, and the “Accessibility at all times” option was chosen by 82% of participants.

**Fig. 3:**
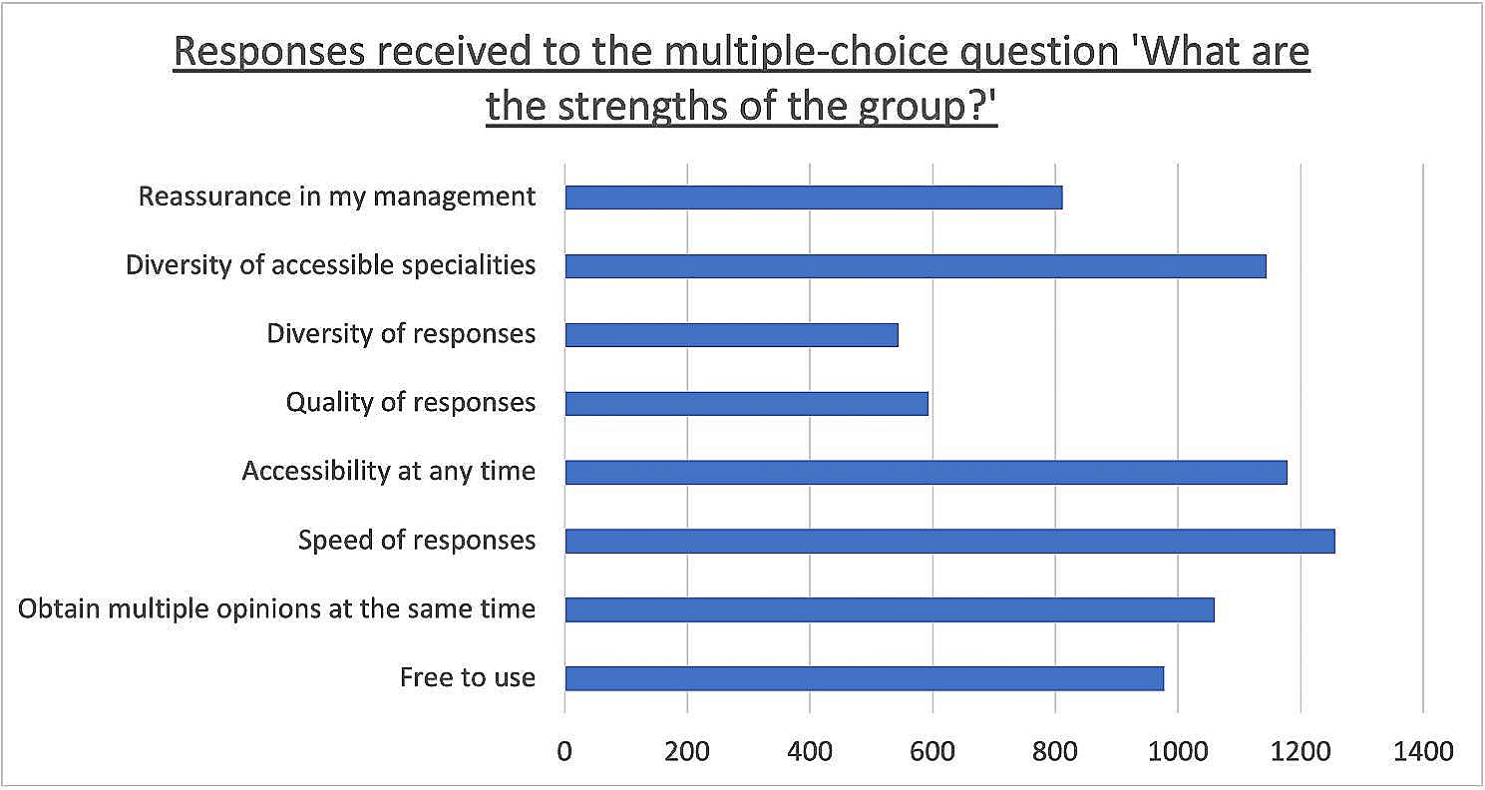
Responses obtained to the multiple-choice question “In your opinion, what are the strengths of the group?“. The results are presented in the number of participants

The detailed results of this question are presented in Fig. 3 and Additional Table 4.

Regarding the multiple-choice question on the group’s weaknesses, the “contradictions between different responses” option was chosen by 51.2% of participants.

**Fig. 4:**
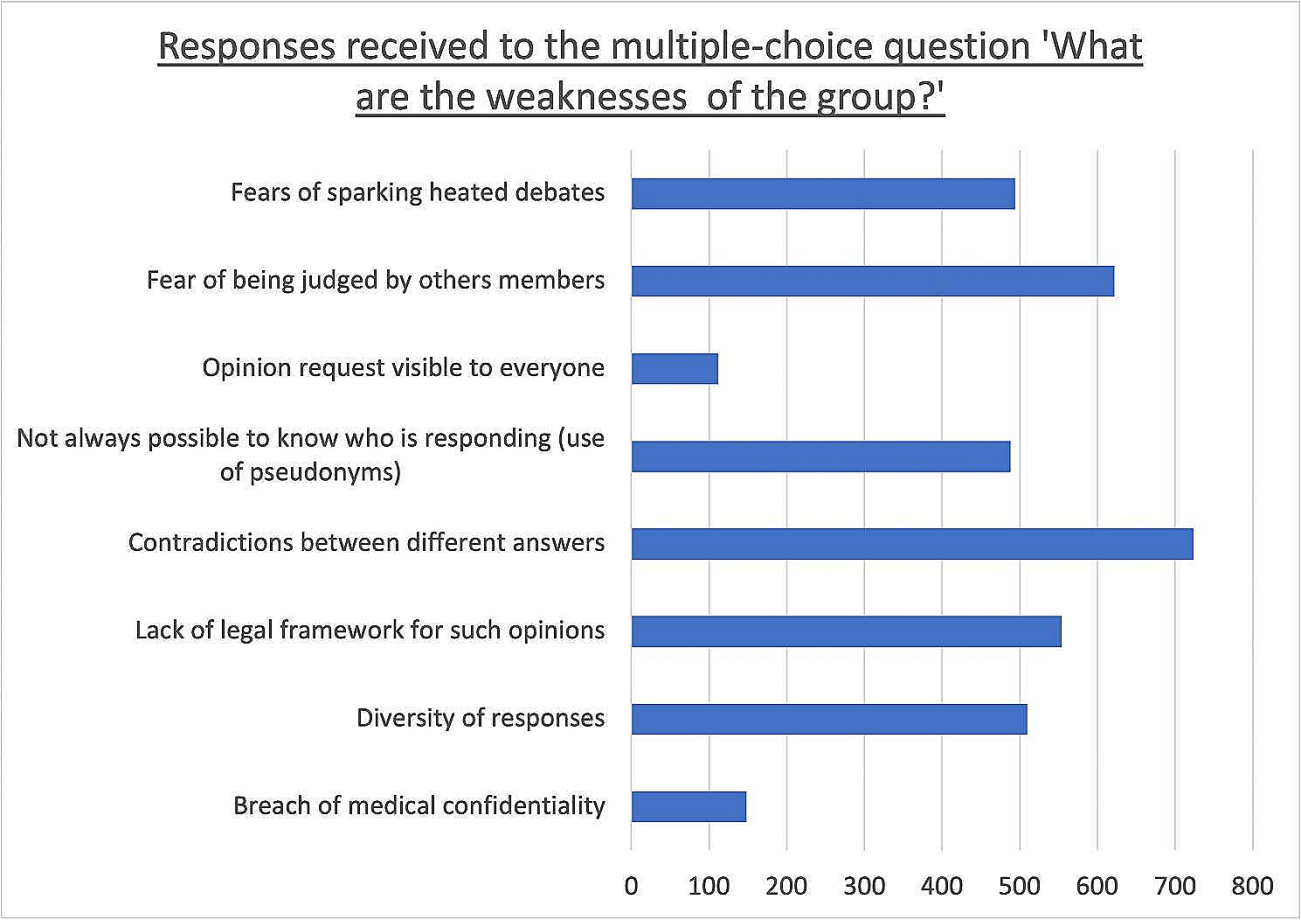
Responses obtained to the multiple-choice question “In your opinion, what are the weaknesses of the group?“. The results are presented in the number of participants

The detailed results of this question are presented in Fig. 4 and Additional Table 5.

### Descriptive analysis: questions regarding medical Pedagogy and continuing medical education (CME)

Regarding the anecdotes, CME points, and documents shared by members, 74.8% of participants gave a score of ≥ 8 (on a scale of 0 to 10, with 0 being “strongly disagree” and 10 being “strongly agree”) for the statement “I have enriched my medical knowledge in several areas.”

Moreover, 54.7% gave a score of ≥ 8 on the same scale for the statement “I have access to documents (recommendations, overviews, practical sheets, etc.) that I would not have had access to without the group.”

**Fig. 5:**
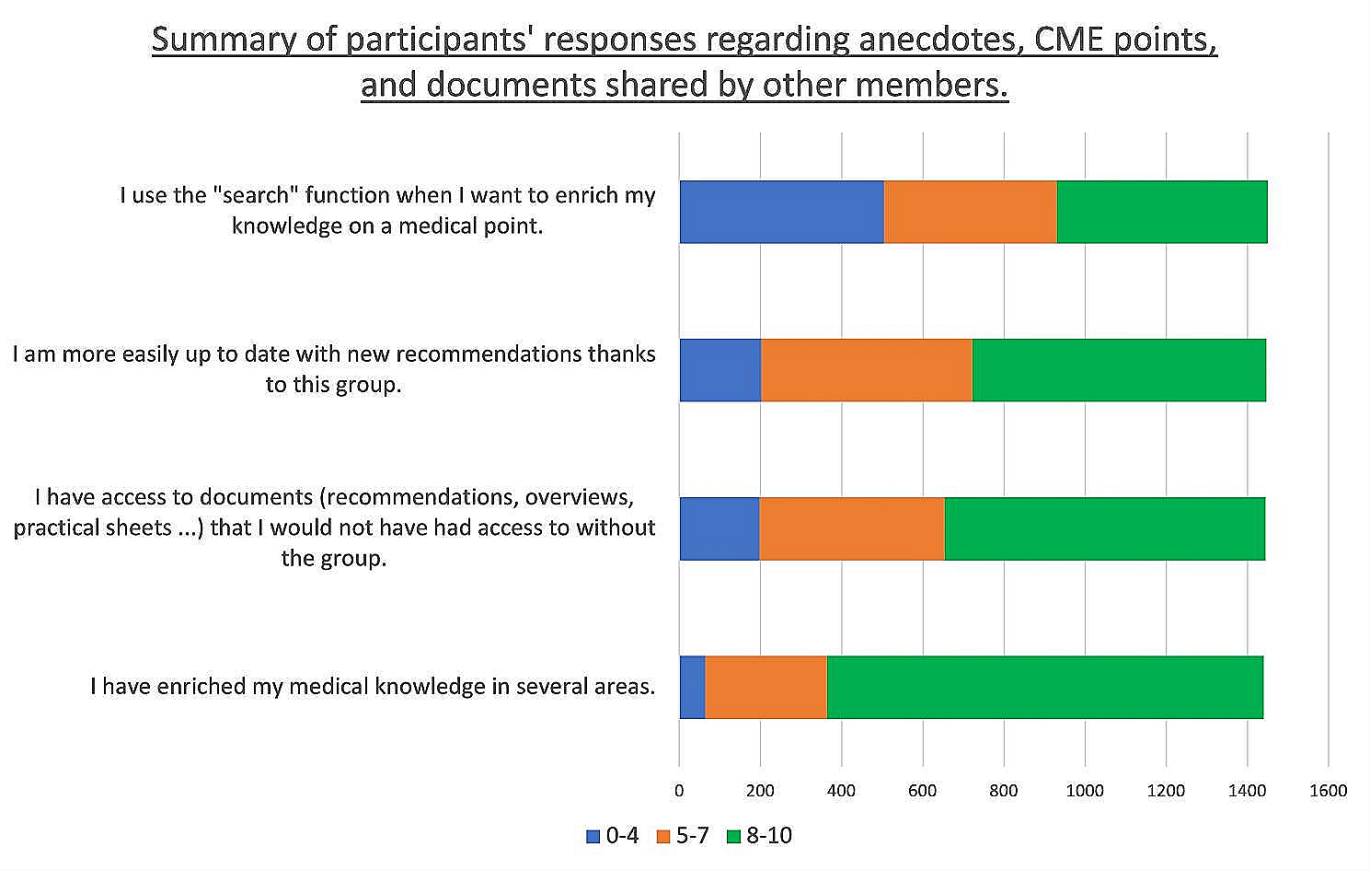
Summary of participants’ responses regarding the anecdotes, CME points, documents shared by other members. Participants were required to give a number between 0 and 10 for each statement, considering 0 as “strongly disagree” and 10 as “strongly agree”. The results are expressed in the number of participants who rated between 0 and 4, between 5 and 7, or 8 and above

The complete results are detailed in Fig. 5 and Additional Table 6.

### Subgroup comparisons

We compared the responses to various questions based on specialty, age, and whether or not the respondent practiced in a medically underserved area.

Regarding the specialty practiced, 70% of general practitioners have previously sought a medical opinion on the group compared to 52.3% for other specialties (*p* < 0.001). Participants practicing in a medically underserved area seek more medical opinions than others (68.4% vs. 61.9%, *p* = 0.02).

The subgroup analysis of responses to the items, based on specialty, whether or not practicing in a medically underserved area, and age, are presented in Table 1.

**Table 1:** Subgroup analysis of responses to items

	n	P value (Chi2 test)
Percentage of participants who have previously requested a medical opinion.		
**Specialty**		< 0,001*
*General practice*	658/940 (70%)	
*Other specialties*	266/508 (52,3%)	
**Exercise in a limited healthcare access**		0,02*
*No*	634/1024 (61,9%)	
*Yes*	286/418 (68,4%)	
**Age**		< 0,001*
*< 30 y.o*	52/134 (38,8%)	
*30–40 y.o*	592/922 (64,2%)	
*40–50 y.o*	188/246 (76,4%)	
*> 50 y.o*	90/144 (62,5%)	
Percentage of participants who gave a score of ≥ 8 for the item'The answers provided reassured me in my management.'		
**Specialty**		< 0,001*
*General practice*	562/666 (84,3%)	
*Other specialties*	206/270 (76,2%)	
**Exercise in a limited healthcare access**		0,42
*No*	528/636 (83,0%)	
*Yes*	236/292 (80,8%)	
**Age**		< 0,001*
*< 30 y.o*	40/50 (80%)	
*30–40 y.o*	518/602 (86,0%)	
*40–50 y.o*	142/192 (74,0%)	
*> 50 y.o*	66/94 (70,2%)	
Percentage of participants who gave a score of ≥ 8 for the item'I have enriched my medical knowledge in several areas.'		
**Specialty**		< 0,001*
*General practice*	736/936 (78,6%)	
*Other specialties*	340/504 (67,5%)	
**Exercise in a limited healthcare access**		0,037*
*No*	744/1018 (73,1%)	
*Yes*	326/416 (78,4%)	
**Age (10-year age brackets)**		0,054
*< 30 y.o*	98/132 (74,2%)	
*30–40 y.o*	706/922 (76,6)	
*40–50 y.o*	176/242 (72,7%)	
*> 50 y.o*	94/142 (66,2%)	
***Age (30-year threshold)***		0,9
*< 30 y.o*	98/132 (74,2%)	
*> 30 y.o*	976/1306 (74,7%)	
Percentage of participants who gave a score of ≥ 8 for the item'I have access to documents (recommendations, summaries, practical sheets, etc.) that I would not have had access to without the group.'		
**Specialty**		< 0,001*
*General practice*	566/936 (60,5%)	
*Other specialties*	224/508 (44,1%)	
**Exercise in a limited healthcare access**		0,038*
*No*	542/1024 (52,9%)	
*Yes*	244/414 (58,9%)	
**Age**		< 0,001*
*< 30 y.o*	62/132 (47,0%)	
*30–40 y.o*	490/922 (53,1%)	
*40–50 y.o*	154/246 (62,6%)	
*> 50 y.o*	84/142 (47,0%)	
Percentage of participants who gave a score of ≥ 8 for the item'I am more easily up to date with new recommendations thanks to this group.'		
**Specialty**		< 0,001*
*General practice*	548/938 (58,4%)	
*Other specialties* **Exercise in a limited healthcare access**	176/508 (34,6%)	0,011*
*No*	490/1024 (47,8%)	
*Yes*	230/416 (55,2%)	
**Age (10-year age brackets)**		
*< 30 y.o*	54/134 (40,3%)	0,085
*30–40 y.o*	464/922 (50,3%)	
*40–50 y.o*	126/246 (51,2%)	
*> 50 y.o*	78/142 (54,9%)	
***Age (30-year threshold)***		0,018*
*< 30 y.o*	54/134 (40,3%)	
*> 30 y.o*	668/1310 (51,0%)	

Results are expressed as the number of participants who responded positively or who gave a score of 8 or higher depending on the item, out of the total number of participants to the question, and in percentage

Statistical analyses were performed using the Chi-squared test. A *p* < 0.05 is considered significant

## Discussion

In France, as of September 1, 2022, the National Council of the Order of Physicians lists 197,811 registered doctors. The Social Networks group studied here has over 22,000 members, representing more than 11% of physicians in France. Our questionnaire was responded to by 1451 participants, which is 6% of the group members. Furthermore, the completion rate is 68.2%. Numerous factors can influence the completion rate, and a good completion rate suggests that the collected data are representative and reliable [11–13]. Our study thus has a significant sample size and demonstrates the value of social networks for the continuing medical education.

The participants in this study are predominantly women (83%), general practitioners (65.1%), and aged between 30 and 40 years (63.8%). Therefore, this cohort is not representative of the French medical population. Indeed, according to data from the Directorate for Research, Studies, Evaluation, and Statistics, in a study published in 2018, among all French doctors, 46% are women, 45% are general practitioners, and the average age is 51 [14]. Our study population is thus much younger and comprises more women and general practitioners. This female predominance aligns with demographic studies on social networks, which show that women, more so than men, use the internet primarily for interpersonal communication and social interactions [15, 16].

Thus, our study population is young and predominantly female, consistent with the demographic that uses social networks.

Regarding requests for medical opinions, our study showed that general practitioners significantly request more opinions than doctors from other specialties. This could be related to the fact that their consultations cover many fields of medicine. A sociological study published in 2008 posited that general practitioners often face a form of uncertainty given the multiple possible diagnoses and having only a limited technical platform at their disposal [17]. This same study highlighted the value of developing care networks or correspondents. Our study emphasized the importance, especially for general practitioners, of feeling less isolated in their medical practice and having quick and easy access to specialized opinions. Our study also showed that requests for opinions are statistically higher among doctors practicing in medically underserved areas, confirming that this Social Networks group helps break the isolation of these doctors, providing easier access to a network of correspondents, virtual as they may be.

Participants indicated that the main advantages of the group are the speed of responses and the group’s accessibility at any time. Moreover, many stated they felt reassured in their patient care approach after seeking an opinion, especially general practitioners, doctors aged 30 to 40, and those under 30, highlighting the group’s value for young doctors starting their careers.

When participants were asked about the group’s drawbacks, the items “contradictions between different responses” and “lack of a legal framework for such opinions” were predominant.

However, our study has a significant selection bias; the participants are primarily the group’s most active members (over 90% of them log into the group more than once a day). Doctors dissatisfied with the group who chose to no longer use it or who rarely log in due to lack of interest had little or no access to the questionnaire. This bias may explain, at least in part, the very positive feedback on the group’s use, as the participants are its most frequent users.

The main risk associated with seeking medical opinions on a Social Networks group is the breach of medical confidentiality. Group members must be particularly vigilant about data anonymization. Images should only be published with the patient’s consent and documented in the medical record. The group’s administrators regularly remind members of these rules to prevent any misconduct. Moreover, the responsibility of the one giving the opinion and the one using it remains unclear.

Regarding the group’s contribution in terms of CME, a majority of participants indicated that they had enriched their knowledge thanks to this group and were more easily updated on new recommendations, especially general practitioners and doctors practicing in medically underserved areas. It should be noted that participants over 30 years old are significantly more likely to report being more easily updated on new recommendations thanks to the group, compared to those under 30. This can be explained by the fact that participants under 30 are mostly residents or clinical leaders, whose training is still very prevalent at this stage of their curriculum.

There is very limited literature concerning the contribution of social networks to physicians’ medical practice. A 2008 study confirmed, after surveying 137 general practitioners, the need for information and CME felt by these doctors [18]. The challenges identified in deepening their knowledge are the lack of time and suitable tools. This Social Networks group thus addresses these two issues, allowing for connection at any time and from anywhere. In our study, participants, especially general practitioners, indicated that they have easier access to documents that enhance their knowledge (HAS recommendations, consensus conferences from learned societies, etc.) and are more easily updated on the latest recommendations thanks to the group.

Thus, this type of group appears to be an effective tool for improving access to medical training. Studies had already shown that social networks could be a tool for CME. For instance, in 2019, a French team published a study analyzing the value of the Twitter® network for CME through the analysis of certain accounts, such as the French pharmacovigilance network, which regularly shares recommendations and quizzes [19]. Another study showed that e-learning is as effective for CME as in-person training and allows for reaching a broader audience [20]. However, these studies refer to official and structured tools (Twitter accounts of an institution, CME organization, etc.) and not to unofficial and unregulated peer groups, as is the case with this Social Networks groups for physicians.

Social Networks, with its accessibility to everyone and ease of use, is a tool that can bring together a wide population. The strength of Social Networks lies particularly in its number of users. To the best of our knowledge, our study is one of the few to address the value of a Social Network group for physician exchange and mutual support. We demonstrate here that physicians, especially general practitioners and those practicing in medically underserved areas, are seeking tools that allow them to seek opinions, discuss patient care, and share recommendations or other training documents. The use of social networks should be expanded, but also secured and structured, to enable physicians to enhance their knowledge.

## Conclusion

In the face of the rapid evolution of digital health, especially highlighted during the Covid crisis, the significance of social networks in medicine cannot be overlooked. Social Networks groups dedicated to physicians not only provide a platform for medical education but also a space for collaborative discussion. The results of our study emphasize the potential of these digital tools, especially for general practitioners and those working in medically underserved areas. As the medical landscape evolves, optimizing the use of these platforms could bolster continuous medical education.

### Electronic supplementary material

Below is the link to the electronic supplementary material.

Supplementary Material 1

Supplementary Material 2

Supplementary Material 3

Supplementary Material 4

Supplementary Material 5

Supplementary Material 6

## Data Availability

No datasets were generated or analysed during the current study.

## Declarations

### Competing of interest

MB, MTD and CB are members of the group studied but have no connection with the group admins. The authors report no competing of interest.
