# Supplementary material for: Evaluating the satisfaction and utility of social networks in medical practice and continuing medical education
Source: BMC Med Educ. 2024 Feb 23;24:186. doi: 10.1186/s12909-024-05149-z (PMC10893748; doi:10.1186/s12909-024-05149-z)
Supplement: Supplementary file 1 — Supplementary Material 1 [file 12909_2024_5149_MOESM1_ESM.docx]

| **Evaluation Questionnaire of the Interest of Social Networks Group for Doctors as a Tool for Medical practice and CME (Continuing Medical Education).** |
| --- |
| 1. **You are :**  - A man - A woman  1. **How old are you ?**  - < 30 y.o - 30-40 y.o - 40-50 y.o - > 50 y.o  1. **What is your medical specialty?**   Dropdown list   1. **What is your main mode of practice?**  - Private practice - Hospital-based - University hospital - Resident - Medical student - Retired - Other: Free field  1. **Where do you practice ?**  - Urban - Semi-rural - Rural  1. **Do you practice in a medical desert (area with limited medical services)?**  - Yes - No  1. **How long have you been in the group?**  - 2017 - 2018 - 2019 - 2020 - 2021 - 2022  1. **ow often do you connect to the group?**  - Once a day or more - Once or twice a week - Once or twice a month - Less than once a month  1. **Have you ever responded to medical opinion requests on the group?**  - Yes, frequently - Yes, occasionally - Yes, rarely - No, never  1. **Have you ever posted a request for a medical opinion?**  - Yes, frequently - Yes, occasionally - Yes, rarely - No, never  1. **If yes, were the answers provided by your colleagues useful to you?**   On a scale of 0 to 10, with 0 being "completely useless" and 10 "completely useful".   1. **If you have ever posted a request for an opinion, do you agree with the following statement: The answers provided allowed me to change my initial prescription/management.**   On a scale of 0 to 10, with 0 being "strongly disagree" and 10 "strongly agree".   1. **If you have ever posted a request for an opinion, do you agree with the following statement: The answers provided are too divergent.**   On a scale of 0 to 10, with 0 being "strongly disagree" and 10 "strongly agree".   1. **If you have ever posted a request for an opinion, do you agree with the following statement: The answers provided helped avoid a diagnostic delay for the patient.**   On a scale of 0 to 10, with 0 being "strongly disagree" and 10 "strongly agree".   1. **If you have ever posted a request for an opinion, do you agree with the following statement: Seeking an opinion on the group sometimes helps avoid sending the patient to the emergency room.**   On a scale of 0 to 10, with 0 being "strongly disagree" and 10 "strongly agree".   1. **If you have ever posted a request for an opinion, do you agree with the following statement: The answers provided allowed me to avoid seeking a specialized opinion.**   On a scale of 0 to 10, with 0 being "strongly disagree" and 10 "strongly agree".   1. **If you have ever posted a request for an opinion, do you agree with the following statement: The answers provided reassured me in my management.**   On a scale of 0 to 10, with 0 being "strongly disagree" and 10 "strongly agree".   1. **If you have ever posted a request for an opinion, do you agree with the following statement: Seeking an opinion on the group makes me feel less isolated in my medical practice.**   On a scale of 0 to 10, with 0 being "strongly disagree" and 10 "strongly agree".   1. **Do you use other platforms for medical opinions, free or paid?**  - Yes - No  1. **If yes, which ones ?**   Free field   1. **In your opinion, what are the strengths of the group? (Multiple answers possible)**  - Free to use - Obtain multiple opinions at the same time - Speed of responses - Accessibility at any time - Quality of responses - Diversity of responses - Diversity of accessible specialities - Reassurance in my management - Other : Free field  1. **What are the weaknesses of the group? (Multiple answers possible)**  - Breach of medical confidentiality - Diversity of responses - Lack of legal framework for such opinions - Contradictions between different answers - Not always possible to know who is responding (use of pseudonyms) - Opinion request visible to everyone - Fear of being judged by others members - Fears of sparking heated debates - Other : Free field  1. **Regarding the anecdotes / CME points / Documents shared by members, do you agree with the following statement: I have enriched my medical knowledge in several areas.**   On a scale of 0 to 10, with 0 being "strongly disagree" and 10 "strongly agree".   1. **Regarding the anecdotes / CME points / Documents shared by members, do you agree with the following statement: I have access to documents (recommendations, overviews, practical sheets ...) that I would not have had access to without the group.**   On a scale of 0 to 10, with 0 being "strongly disagree" and 10 "strongly agree".   1. **Regarding the anecdotes / CME points / Documents shared by members, do you agree with the following statement: I am more easily up to date with new recommendations thanks to this group.**   On a scale of 0 to 10, with 0 being "strongly disagree" and 10 "strongly agree".   1. **Regarding the anecdotes / CME points / Documents shared by members, do you agree with the following statement: I use the "search" function when I want to enrich my knowledge on a medical point.**   On a scale of 0 to 10, with 0 being "strongly disagree" and 10 "strongly agree".   1. **Do you have any comments?**   Free field |

Additional Table 1: Evaluation Questionnaire of the Interest of a Social Network Group for Doctors as a Tool for Medical practice and CME (Continuing Medical Education).
