# Supplementary material for: Evaluating the satisfaction and utility of social networks in medical practice and continuing medical education
Source: BMC Med Educ. 2024 Feb 23;24:186. doi: 10.1186/s12909-024-05149-z (PMC10893748; doi:10.1186/s12909-024-05149-z)
Supplement: Supplementary file 2 — Supplementary Material 2 [file 12909_2024_5149_MOESM2_ESM.docx]

| **Gender**  *Women*  *Men* | *Total number of responses : 1444*  1198 (83%)  246 (17%) |
| --- | --- |
| **Age**  *<30 y.o*  *30-40 y.o*  *40-50 y.o*  *>50 y.o* | *Total number of responses : 1448*  134 (9,3%)  924 (63,8%)  246 (17%)  144 (9,9%) |
| **Medical specialty**  *General practice*  *Other specialties* | *Total number of responses : 1444*  940 (65,1%)  504 (34,9%) |
| **Mode of practice**  *Private practice*  *Hospital-based*  *University hospital*  *Resident*  *Other (medical student, retired …)* | *Total number of responses : 1446*  862 (59,6%)  244 (16,9%)  76 (5,3%)  74 (5,1%)  190 (13,1%) |
| **Place of exercise**  *Urban*  *Semi-rural*  *Rural* | *Total number of responses : 1446*  846 (58,5%)  438 (30,3%)  162 (11,2%) |
| **Exercise in a limited healthcare access**  *Yes*  *No* | *Total number of responses : 1444*  418 (28,9%)  1026 (71,1%) |
| **Frequency of connexion**  *Once a day or more*  *Once or twice a week*  *Once or twice a month*  *Less than once a month* | *Total number of responses : 1446*  1312 (90,7%)  122 (8,4%)  6 (0,4%)  6 (0,4%) |

Additional Table 2: Characteristics of the study participants.

*The results are presented in terms of the number of participants and in percentage.*
