# Supplementary material for: Evaluating the satisfaction and utility of social networks in medical practice and continuing medical education
Source: BMC Med Educ. 2024 Feb 23;24:186. doi: 10.1186/s12909-024-05149-z (PMC10893748; doi:10.1186/s12909-024-05149-z)
Supplement: Supplementary file 3 — Supplementary Material 3 [file 12909_2024_5149_MOESM3_ESM.docx]

| **The answers provided allowed me to change my initial prescription/management.**  *0-4*  *5-7*  *≥ 8* | *Total number of responses : 948*  102 (10,8%)  352 (37,1%)  494 (52,1%) |
| --- | --- |
| **The answers provided are too divergent.**  *0-4*  *5-7*  *≥ 8* | *Total number of responses : 942*  582 (61,8%)  278 (29,5%)  82 (8 ,7%) |
| **The answers provided helped avoid a diagnostic delay for the patient.**  *0-4*  *5-7*  *≥ 8* | *Total number of responses : 924*  214 (23,2%)  330 (35,7%)  380 (41,1%) |
| **Seeking an opinion on the group sometimes helps avoid sending the patient to the emergency room.**  *0-4*  *5-7*  *≥ 8* | *Total number of responses : 924*  278 (30,1%)  352 (38,1%)  294 (31,8%) |
| **The answers provided allowed me to avoid seeking a specialized opinion.**  *0-4*  *5-7*  *≥ 8* | *Total number of responses : 934*  178 (191%)  330 (35,3%)  426 (45,6%) |
| **The answers provided reassured me in my management.**  *0-4*  *5-7*  *≥ 8* | *Total number of responses : 940*  28 (3,0%)  144 (15,3%)  768 (81,7%) |
| **Seeking an opinion on the group makes me feel less isolated in my medical practice.**  *0-4*  *5-7*  *≥ 8* | *Total number of responses : 934*  44 (4,7%)  90 (9,6%)  800 (85,7%) |

Additional Table 3: Summary of participants' responses regarding the feedback provided to medical inquiries.

*Participants who had previously sought medical advice in the group were required to give a number between 0 and 10 for each statement, considering 0 as "strongly disagree" and 10 as "strongly agree".*

*The results are expressed as the number of participants over the total number of responses and as a percentage of participants who scored between 0 and 4, between 5 and 7, or 8 and above.*
