# Supplementary material for: Evaluating the satisfaction and utility of social networks in medical practice and continuing medical education
Source: BMC Med Educ. 2024 Feb 23;24:186. doi: 10.1186/s12909-024-05149-z (PMC10893748; doi:10.1186/s12909-024-05149-z)
Supplement: Supplementary file 4 — Supplementary Material 4 [file 12909_2024_5149_MOESM4_ESM.docx]

|  | *Total number of responses : 1011* |
| --- | --- |
| Free to use | 978 (68,1%) |
| Obtain multiple opinions at the same time | 1060 (73,8%) |
| Speed of responses | 1256 (87,5%) |
| Accessibility at any time | 1178 (82%) |
| Quality of responses | 594 (41,4%) |
| Diversity of responses | 544 (37,9%) |
| Diversity of accessible specialities | 1144 (79,7%) |
| Reassurance in my management | 812 (56,5%) |

Additional Table 4: Responses obtained to the multiple-choice question "In your opinion, what are the strengths of the group?".

*The results are expressed in terms of the number of participants who selected the item and in percentage.*
