# Supplementary material for: Evaluating the satisfaction and utility of social networks in medical practice and continuing medical education
Source: BMC Med Educ. 2024 Feb 23;24:186. doi: 10.1186/s12909-024-05149-z (PMC10893748; doi:10.1186/s12909-024-05149-z)
Supplement: Supplementary file 5 — Supplementary Material 5 [file 12909_2024_5149_MOESM5_ESM.docx]

|  | *Total number of responses: 989* |
| --- | --- |
| Breach of medical confidentiality | 148 (10,5%) |
| Diversity of responses | 510 (36,1%) |
| Lack of legal framework for such opinions | 554 (39,2%) |
| Contradictions between different answers | 724 (51,2%) |
| Not always possible to know who is responding (use of pseudonyms) | 488 (34,5%) |
| Opinion request visible to everyone | 112 (7,9%) |
| Fear of being judged by others members | 622 (44%) |
| Fears of sparking heated debates | 494 (34,9%) |

Additional Table 5: Responses obtained to the multiple-choice question "In your opinion, what are the weaknesses of the group?".

*The results are expressed in terms of the number of participants who selected the item and in percentage.*
