# Supplementary material for: Evaluating the satisfaction and utility of social networks in medical practice and continuing medical education
Source: BMC Med Educ. 2024 Feb 23;24:186. doi: 10.1186/s12909-024-05149-z (PMC10893748; doi:10.1186/s12909-024-05149-z)
Supplement: Supplementary file 6 — Supplementary Material 6 [file 12909_2024_5149_MOESM6_ESM.docx]

| **I have enriched my medical knowledge in several areas.**  *0-4*  *5-7*  *≥ 8* | *Total number of responses : 1440*  64 (4,4%)  300 (20,8%)  1076 (74,8%) |
| --- | --- |
| **I have access to documents (recommendations, overviews, practical sheets ...) that I would not have had access to without the group.**  *0-4*  *5-7*  *≥ 8* | *Total number of responses : 1444*  198 (13,7%)  456 (31,6%)  790 (54,7%) |
| **I am more easily up to date with new recommendations thanks to this group.**  *0-4*  *5-7*  *≥ 8* | *Total number of responses : 1446*  202 (14,0%)  520 (36,0%)  724 (50,0%) |
| **I use the "search" function when I want to enrich my knowledge on a medical point.**  *0-4*  *5-7*  *≥ 8* | *Total number of responses : 1450*  504 (34,7%)  426 (29,4%)  520 (35,9%) |

Additional Table 6: Summary of participants' responses regarding anecdotes, Continuing Medical Education (CME) points, and documents shared by other members.

*Participants were required to give a number between 0 and 10 for each statement, considering 0 as "strongly disagree" and 10 as "strongly agree".*

*The results are expressed as the number of participants and as a percentage of participants who scored between 0 and 4, between 5 and 7, or 8 and above.*
